# Integrated information storage and transfer with a coherent magnetic device

**DOI:** 10.1038/srep13665

**Published:** 2015-09-08

**Authors:** Ning Jia, Leonardo Banchi, Abolfazl Bayat, Guangjiong Dong, Sougato Bose

**Affiliations:** 1 State key laboratory of precision spectroscopy, Department of Physics, East China Normal University, Shanghai 200062, China; 2 Department of Physics and Astronomy, University College London, Gower Street, WC1E 6BT London, United Kingdom

## Abstract

Quantum systems are inherently dissipation-less, making them excellent candidates even for classical information processing. We propose to use an array of large-spin quantum magnets for realizing a device which has two modes of operation: memory and data-bus. While the weakly interacting low-energy levels are used as memory to store classical information (bits), the high-energy levels strongly interact with neighboring magnets and mediate the spatial movement of information through quantum dynamics. Despite the fact that memory and data-bus require different features, which are usually prerogative of different physical systems – well isolation for the memory cells, and strong interactions for the transmission – our proposal avoids the notorious complexity of hybrid structures. The proposed mechanism can be realized with different setups. We specifically show that molecular magnets, as the most promising technology, can implement hundreds of operations within their coherence time, while adatoms on surfaces probed by a scanning tunneling microscope is a future possibility.

The ultimate fate of the miniaturization of information processing devices naturally leads to the quantum regime even for realizing classical computers. Although quantum computation holds the promise to be the next step in the evolution of information technology, quantum algorithms have been proved to speed up only very specific computational tasks, notably prime factorization^1^ and database search^2^. Moreover, quantum mechanical systems are, in principle, based on unitary operations which are reversible and thus dissipation-less. In view of this, using a quantum device might also provide a solution to certain technological obstacles in classical information technology, *e.g.* heat production. Such information processing is also less demanding with respect to quantum coherence and their realization is thus less challenging. Additionally the miniaturization of electronics (even in the context of classical computation) with the demands of more data and functional density naturally leads to the quantum world.

Any information processing device essentially needs at least two different units to operate properly, namely a long-lived memory and a fast data-bus for communication between different registers or processors. The cells of the memory unit have to be well isolated from the rest of the system. There are several atomic scale spin systems which show suitable properties for operating as memories, such as nitrogen vacancy centers in diamond^3^, nuclear spins in solid state systems^4^,^5^, molecular magnets^6^ and adatoms on surfaces^7^,^8^,^9^,^10^. In contrast, for information transfer, a strong interaction between the cells of the data-bus is required for fast operation within the coherence time. Good examples of quantum systems with strong interactions includes, ion traps^11^, superconducting qubits^12^, electronic spins in gated quantum dots^13^,^14^, and donors in silicon^15^,^16^. The opposite demands for isolated memory and strongly interacting data-bus units make it notoriously difficult to implement both units in the same physical device. While hybrid structures (e.g. atom-photon, superconducting qubits-microwave, nuclear-electronic spin) have been proposed^17^,^18^,^19^ for fulfilling this task, a very high degree of precision is needed to control two different physical systems and their interaction, in order to transfer information from one system to another.

In this work we show that nano-magnets with a large half-integer spin momentum can simultaneously act both as memory and data-bus for information transfer within the same setup without the complexity of hybrid structures. In fact, the spin levels in neighboring sites can interact through different mechanisms resulting in exchange interactions which may vary several orders of magnitude between different spin levels. In large-spin systems, we propose to use the flexibility in selecting two weakly interacting low-energy spin levels for encoding a classical bit, while strongly interacting high-energy spin levels, act quantum mechanically for transferring such information between distant memory cells. Information transfer between the two units, namely the high and low energy subspaces, is achieved via global electromagnetic pulses acting on the whole system.

We specifically consider an array of high-spin magnets which interact through the Heisenberg Hamiltonian with a large zero-field energy splitting^20^. We show that such a large energy-level separation together with the inherent selection rule determined by the interaction results in different effective exchange interaction for the low- and high-energy subspaces. This in turn implies that the low-energy levels display a weak effective interaction, making them suitable for storage, while high-energy levels result in a strong effective interaction which can be exploited for fast information transmission. Although higher spin systems have been proposed for quantum communication^21^,^22^,^23^,^24^, neither of them can implement both the memory and data-bus. Our proposed mechanism can be realized in different physical implementations of high-spin magnets with large zero-field splitting. This includes magnetic adatoms on surfaces^25^, donors on silicon^15^,^16^ and molecular magnets^26^,^27^,^28^,^29^,^30^. We specifically consider the latter as a testbed for implementing our proposal. Indeed, molecular magnets have recently attracted lot of attentions thanks to the flexibility in engineering their properties through chemical synthesis^27^,^28^ and their long-coherence time^29^,^30^. Our proposal, is fully accessible to current technology and allows for hundreds of operations using the same parameters achieved in recent experiments^27^,^29^,^30^ within the coherence time of the system.

## Introducing the Model

We consider a one dimensional system composed of *N* quantum nanomagnets with a certain spin *S*. The magnetic interaction is described by the Hamiltonian


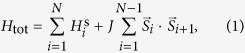


where *J* is the strength of the isotropic exchange interaction between magnets and 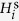 is the local Hamiltonian acting on the *i*th magnet. As a paradigmatic model we consider


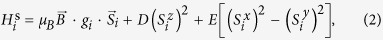


where 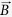 is the magnetic field, *g*_*i*_ is the position dependent Landé *g*-factor, *μ*_*B*_ is the Bohr magneton, *D* models the zero field splitting, and *E* represents the planar anisotropy in the crystal field interaction. For the moment we consider no applied magnetic field, so 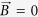.

**Figure 1:**
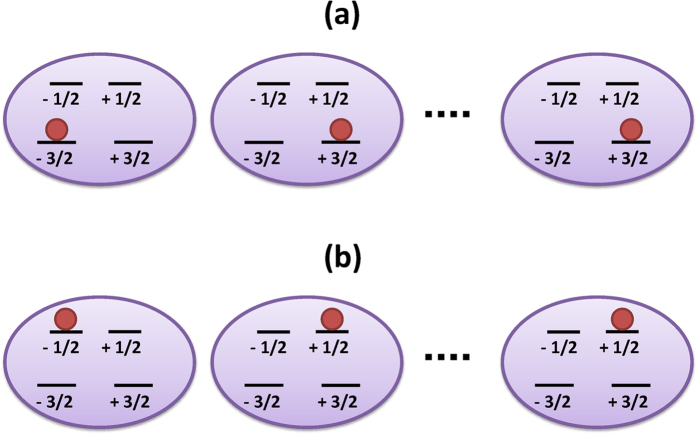
Low and high energy subspaces. Array of *S* = 3/2 spins with large negative zero-field splitting *D* initialized in: **(a)** the low-energy subspace in the state of 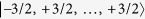; **(b)** high-energy subspaces in the state of 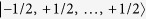. The transition between the low and high energy subspaces can be achieved with a global resonant pulse.

When *S* is half integer the eigenstates of (2) comes into pair of degenerate levels (called Kramers doublet^20^) with opposite magnetization *m* along the *z* direction (see Fig. 1). The states 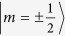, which are the stable states when *D* > 0, are not suitable to implement a memory because a magnetic field, whatever small, can induce a transition between them. On the other hand, the states 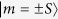, which are the stable states when *D* < 0, represent a good candidate to implement a classical bit in a quantum memory because there is no direct physical coupling between them. In fact, since a jump between these two states can only occur via multiple-step processes, bit flip errors are exponentially suppressed.

## Effective Dynamics in the Low-energy and High-energy Subspaces

In this section we prove that the low-energy Hilbert space (that we call memory subspace) 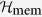 is suitable to store information, while the high energy Hilbert space 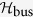 can be used to implement a data-bus for fast information transfer between remote memory cells. We start our analysis by deriving two effective Hamiltonians respectively within the two subspaces. For the moment we concentrate on *S* = 3/2 spin systems (though later in the paper we will extend our analysis to higher spin systems) where


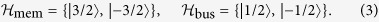


**Figure 2:**
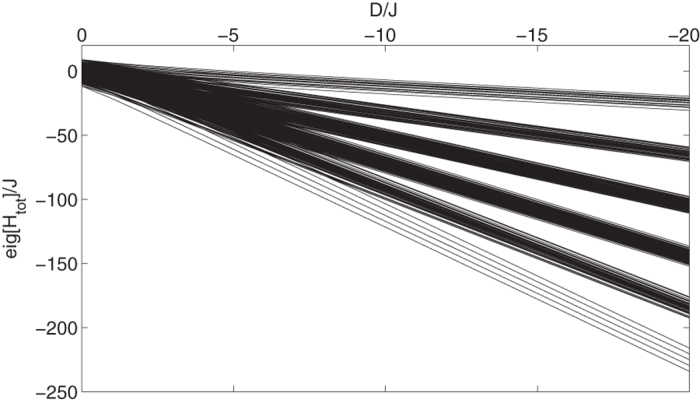
Emergence of energy bands. Energy spectrum of *H*_tot_ for *N* = 5, 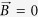, and *E* = 0, as a function of *D*/*J*. When |*D*| ≫ *J* a band structure appears in the spectrum. The lowest energy band (composed by the 2^*N*^ levels in the subspace 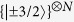) forms the memory subspace, while the highest energy band (composed by the 2^*N*^ levels in the subspace 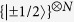) forms the data-bus subspace.

For negative *D*, in the regime |*D*| ≫ *J*, these two effective subspaces become energetically well separated. To see this, in Fig. 2 we plot the spectrum of *H*_tot_ as a function of *D*. As it is evident from the figure, a band structure appears when |*D*| ≫ *J* in which the lowest band is formed by states in the memory subspace 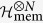, while the highest band is formed by states in the data-bus subspace 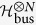. If we initialize our systems in one the bands, throughout the dynamics other bands are hardly populated. This suggests that there should be an effective description for the dynamics within the memory and data-bus subspaces. In the next section we provide effective Hamiltonians for each of these subspaces.

### Spin dynamics in the low-energy subspace

We now consider the regime where *D* < 0 and |*D*| ≫ *J* while 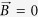. In this regime the states 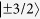 are degenerate and well separated from the states 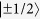. This allows us to get an effective interaction between the low energy states 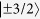 which is mediated through a “virtual” coupling with the high energy states.

**Figure 3:**
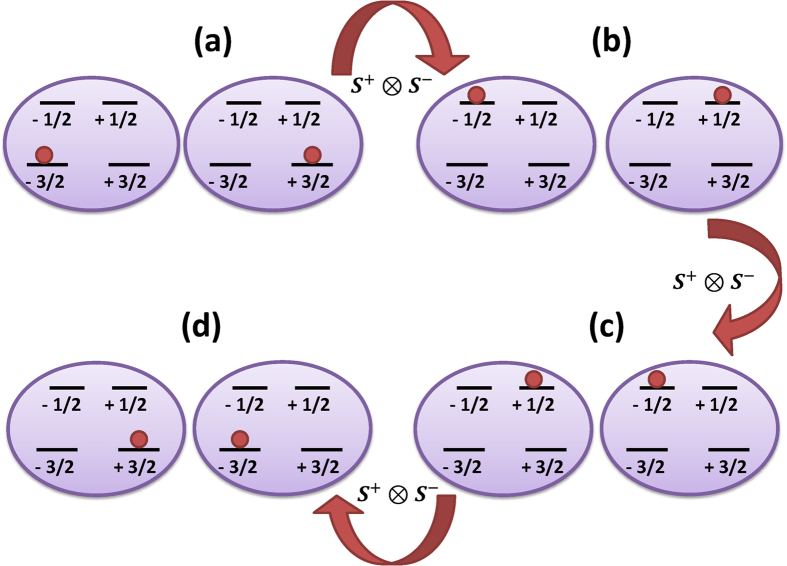
The third order effective hopping term in the memory-subspace. The third-order effective hopping Hamiltonian in the low-energy subspace can be explained by the application of 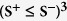 (with *S*^±^ = *S*^*x*^ ± *iS*^*y*^), which arises in the third order perturbation theory used for getting the effective Hamiltonian (see Supplementary Material for more details). In fact, the three consecutive operations of the term 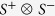 result in spin swap in the low-energy subspace through virtually populating the high-energy states. We show the states 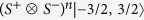, for *n* = 0 (**a**), *n* = 1 (**b**), *n* = 2 (**c**), *n* = 3 (**d**), which are populated during the process.

We derive the effective Hamiltonian using the theory presented in Supplementary Material, which is based on two key assumptions: (i) large energy separation (≈2*D*) between the states 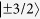 and the states 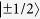; (ii) no initial population of the states 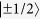. We found that up to the second order in *J*/*D* and *E*/*D* one gets


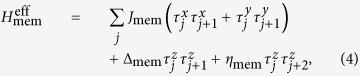


where,


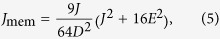



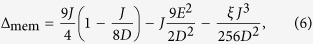



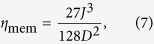


and *ξ* = 90 in the bulk and *ξ* = 63 at the boundaries. In (4) the matrices *τ*_*x*,*y*,*z*_ are Pauli operators defined in the effective subspace 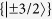. To the lowest order the effective interaction in the low-energy subspace is of Ising-type, as shown also in^31^, and thus does not induce direct transitions between energy levels. Magnetic exchange between two neighboring sites is governed by a third order effect, as reflected in the effective coupling *J*_mem_ displayed in Eq. (5). This third order process is mediated by the virtual processes depicted in Fig. 3 where two high-energy levels are populated.

Since the exchange is only a third order process in *J*/*D*, an eventual magnetic transfer between neighboring sites would take place in the large time scale of 1/*J*_mem_. Those transfer mechanisms can thus be ignored (for suitably large *D*) in all processes that are governed by lower order mechanisms, such as the transfer in the higher energy subspace that we will discuss later. Hence, it is legitimate to use this subspace as a memory for storing information.

Notice that the difference between ferromagnetic and anti-ferromagnetic couplings, *i.e.* the sign of *J*, does not alter the results of our proposal, as the system is not initialized in the ground state.

### Spin dynamics in the high-energy subspace

We now consider the scenario in which the system is prepared in the high energy subspace spanned by 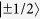. We again apply the partial integration technique, described in the Supplementary Material. The resulting effective Hamiltonian to the first order in *J*/*D* is


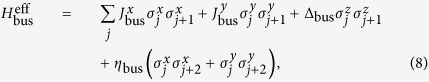


where the matrices *σ*_*x*,*y*,*z*_ are the Pauli operators defined in the effective subspace 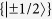, and


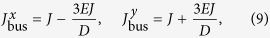



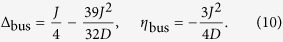


**Figure 4:**
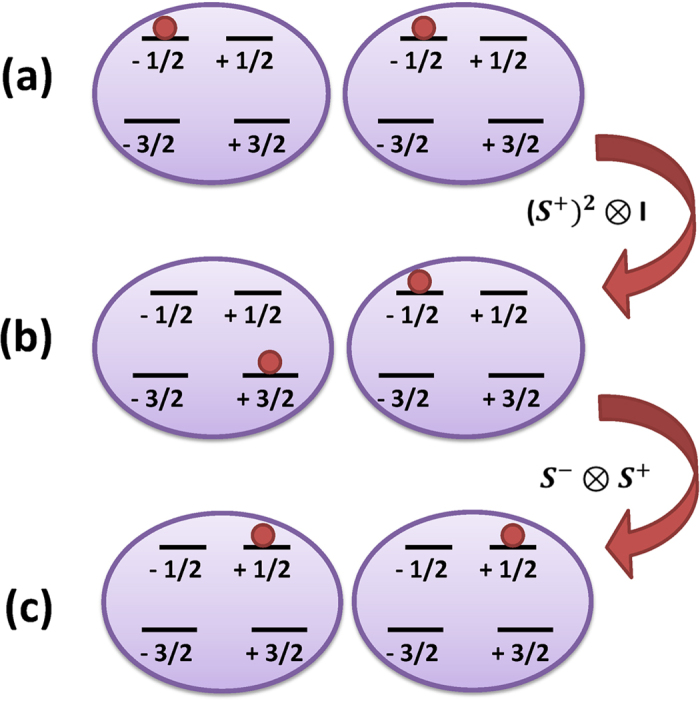
The second order asymmetry term in the effective data-bus Hamiltonian. In the presence of in-plane anisotropy (i.e. *E* > 0) the spin exchange couplings 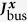 and 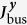 become asymmetric in the *x* and *y* directions, as given in the effective Hamiltonian of Eq. (8). The origin of this asymmetry is a second order process through which the action of in-plane anisotropy 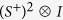 (or its conjugate 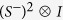) followed by the operation of the usual spin exchange 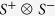 results in a term like 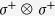 (or equivalently 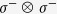) in the effective Hamiltonian of the high-energy subspace. We show the states (**a**) 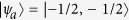, (**b**) 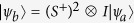, (**c**) 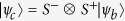, which are populated during the third order process.

As it is evident from the above formulae, *J*_*x*_ = *J*_*y*_ = *J* to the zeroth order, while there is a first order anisotropy in the *xy* plane caused by the crystal field anisotropy *E*. The origin of this effective anisotropy is schematically explained in Fig. 4 in which one state in the low energy subspace is virtually populated.

To see the performance of bit transfer in the data-bus subspace in a chain of *N* magnets we compute the fidelity


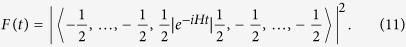


**Figure 5:**
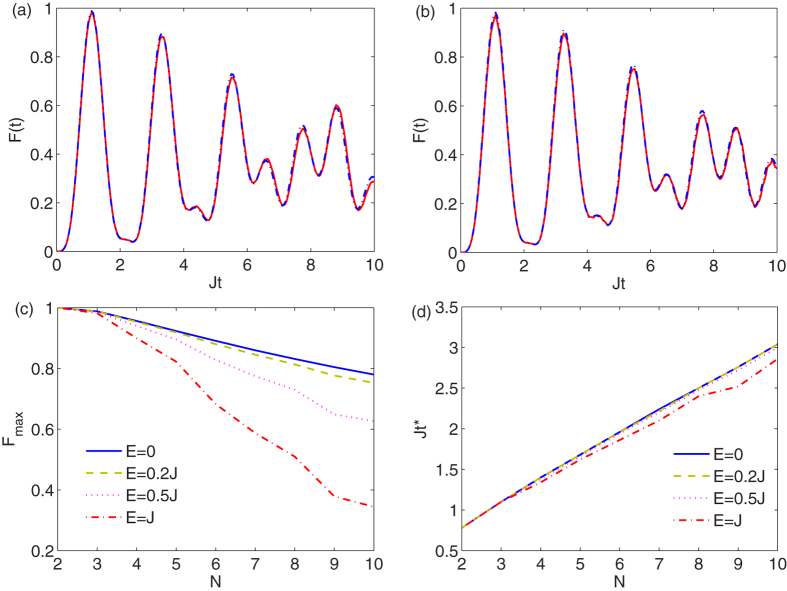
Dynamics in high energy subspace. (**a,b**) Fidelity *F*(*t*), given in Eq. (11), evaluated with the real Hamiltonian (dashed blue curve) and the effective Hamiltonian (solid red curve) acting on the data-bus subspace 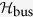. The chain of length is *N* = 3 and the parameters are *D* = −20*J* and *E* = 0 for (**a**) and *E* = *J* for (**b**). (c) The maximum of bit transfer fidelity *F*_*max*_ as a function of length *N* for *D* = −20*J* and different values of *E*. (**d**) Scaling of the transfer time t^*^ as a function of the length *N*, using the same parameters of (**c**).

We first calculate the fidelity with the Hamiltonian *H* = *H*_tot_, to take account of the influence of the lower-energy subspace on the information transfer in higher energy space. We also compute the fidelity with 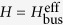 to check the validity of the effective Hamiltonian 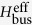 in higher-energy subspace. We make a comparison of the time evolutions of the fidelity computed for the total Hamiltonian *H*_tot_ and the effective Hamiltonian 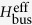 for a spin chain of *N* = 3 with the parameters *D* = −20*J* and *E* = 0 and *J* respectively in Fig. 5(a,b). The perfect match of the two curves in Fig. 5(a,b) shows negligible influence of the lower-energy subspace on transfer of the information initially written in the higher-energy subspace along the nano-magnete chain, and thus the higher-energy subspace could function as a data-bus for quantum information transfer.

In the high-energy subspace two neighboring spins are directly coupled by the Heisenberg interaction (1). This results in a very large exchange coupling, (*i.e. J*_bus_ ≈ *J*) which, in turns, implies a very fast transfer dynamics compared with the low energy subspace. As *J*_bus_/*J*_mem_ ≈ (*D*/*J*)^2^, one can see that in the regime where the effective Hamiltonian picture is valid (say *D* ≥ 10*J*), the high energy subspace is faster by at least two orders of magnitude. This justifies the use of the high-energy subspace for computational tasks with a fast bit transmission.

To quantify quality of the bit transfer one can consider the time *t* = t^*^ at which the fidelity *F*(*t*), given in Eq. (11), peaks for the first time and takes the value *F*_max_ = *F*(t^*^). In fact, in real systems, decoherence deteriorates the quality of bit-transfer and it is unwise to wait for later peaks. To see the scalability of the bit transfer in larger chains we plot *F*_max_, using the effective Hamiltonian (8), as a function of length *N* in Fig. 5(c). Although due to the non-linear dispersion relation, the maximum fidelity *F*_max_ decreases with increasing *N*, it still remains above 0.75 for *N* ≤ 10. Moreover, in Fig. 5(c) we see that the transverse anisotropy *E* has always a detrimental role for transmission, therefore those systems with vanishing *E* are preferable. It is worth emphasizing that the effective Hamiltonian description is always valid for all *N* provided that |*D*| ≫ *J*. In view of this, the decrease of *F*_max_ as a function of *N* is only due to the dispersive dynamics of the effective Hamiltonian (see *e.g.*^32^). In order to improve the fidelity for larger values of *N* one has to linearize the dispersion relation through local engineering of the system parameters (such as the exchange coupling *J*), as extensively discussed in the literature^33^. As shown in Fig. 5(d), the optimal time t^*^ linearly increases by *N* but still is, at least, one order of magnitude faster than the time scale of bit swap in the memory subspace, given by 1/*J*_mem_.

Note that the distance over which a bit can be moved, strictly speacking, needs not be limited. For example we can adapt some minimal engineering techniques to move bits over much longer distances^34^, though here the Hamiltonian is rather different.

### Transitions between memory and data-bus subspaces

We now study how one can simultaneously transfer all the states from the low-energy to the high-energy subspace and *vice versa*. When the chain is composed of a single nanomagnet, the memory (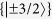) and bus 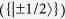 Hilbert subspaces have an energy separation of 2*D*. A resonant spin transition can be obtained with an electromagnetic pulse 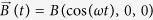 with *ω* ≃ 2*D*. This physically motivated intuition can be made more rigorous. Indeed, near resonance, the time-dependent interactions can be approximated in the rotating picture with a time-independent Hamiltonian, and the off resonant energy levels are then traced out using theory presented in the Supplementary Material. We found that in the limit 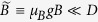 a transition with Δ*S*^*z*^ = ±1 occurs with a transition time 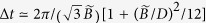 and a transition fidelity 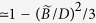. Therefore, in the limit *μ*_*B*_* gB* ≪ *D* the transition fidelity is almost one, as it has been proved by our numerical simulations (not shown here).

On the other hand, in a many nanomagnet scenario the pulse has to be fast enough to neglect the interaction *J* between neighboring magnets, so the optimal working regime is *J* ≪ *μ*_*B *_*gB* ≪ *D*.

### Higher spin systems

The procedure described above can be applied also for higher half-integer spin *S* systems. In fact, the dynamics in the high energy subspace 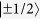 is still governed by an effective Hamiltonian which has a similar form of Eq. (8). In particular, the leading term is an exchange coupling which results in fast transmission times (≈1/*J*). In contrast, the storage quality of the memory subspace 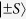 is significantly improved as *S* increases. Indeed, the effective coupling *J*_mem_ ∝ *J*(*J*/*D*)^(2*S*−1)^ becomes smaller by increasing *S*, making the storage much less prone to errors over a longer time scale. However, in some higher-spin systems, like rare-earth ions, there might be higher order anisotropy terms (i.e. Stevens operators) in the Hamiltonian^35^,^36^ which might change the effective couplings.

In addition, bit-flip errors, i.e. spurious transitions between the states 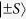, are exponentially suppressed as they require multiple jumps through higher energy levels. This, however, comes with the price that a transition from the memory to the data-bus subspaces demands multiple pulse sequences (namely (2*S* − 1)/2 consecutive pulses) which increase the complexity of the process.

## Imperfections

In this section we consider two sources of imperfections which may affect our protocol, namely decoherence and possible long-range interactions arising, *e.g.*, from dipolar couplings between distant nanomagnets.

### Effect of decoherence

In a real physical system it is notoriously difficult to keep the system isolated from the surrounding environment. Depending on the nature of the interaction between the system and the environment one may have different decoherence processes. In particular, for the main target experiments of our theoretical proposal, e.g. molecular magnets and magnetic adatoms on surfaces, the dissipation time *T*_1_ is larger than the dephasing time *T*_2_ by several order of magnitudes. Dephasing, which is thus the main source of decoherence, arises because of complicated interactions with other degrees of freedom. In this paper we consider a simple model of decoherence, i.e. caused by random energy level fluctuations due to nearby magnetic and electric impurities. By averaging over the possible random time fluctuations one obtains a master equation for the evolution of the system which has the Lindblad form^8^


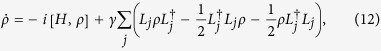


where *ρ*(*t*) is the system density matrix, *γ* is the dephasing rate and *L*_*j*_ are Lindblad operators. Due to the fact that *T*_1_ ≫ *T*_2_, we only consider dephasing here, which can be modeled with 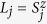. Although the master equation (12) neglects non-Markovian effects, it is widely used to model qualitatively the action of the environment in the type of systems that we consider for physical realization^8^.

**Figure 6:**
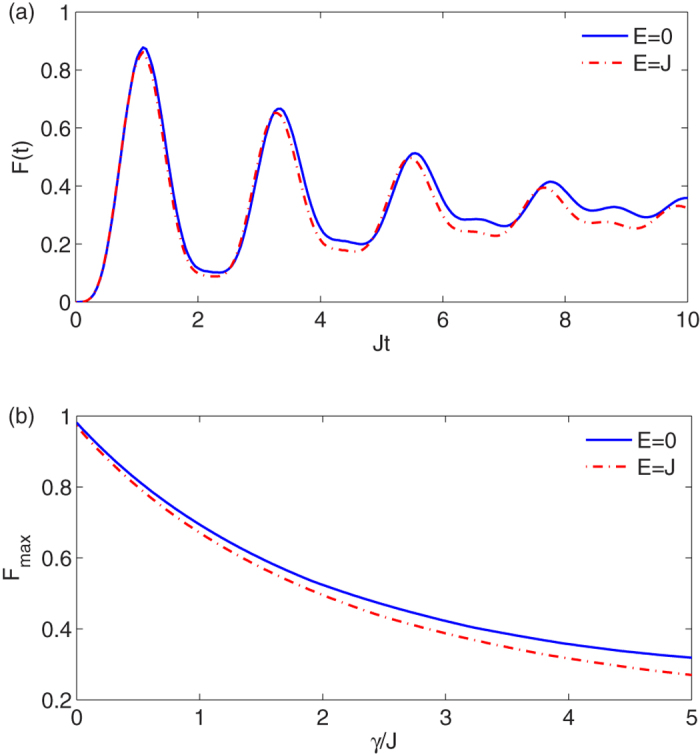
Decoherence in high energy subspace. (**a**) The bit-tranfer fidelity *F*(*t*) in the data-bus subspace with dephasing rate *γ* = 0.5*J* when *D* = −20*J* and *N* = 2. The chosen value for *γ* is extremely pessimistic even for larger chains, and we have chosen this value in order to show the decay in shorter time scales. (**b**) The maximum fidelity *F*_*max*_ as a function of *γ* for two different values of anisotropy *E*, when *D* = −20*J* and *N* = 2.

We first consider the case where the system is prepared in the high-energy subspace for computational tasks and we study the effect of dephasing on the fidelity of state transmission. As an example the two-site system is initialized in the pure state 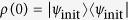, where 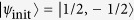, but because of the non-unitary evolution (12) it evolves into a mixed state *ρ*(*t*). The resulting fidelity of state swap is therefore 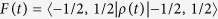. In Fig. 6(a) we plot *F*(*t*) as a function of time for a very strong *γ* = 0.5*J*. We have chosen a high value of *γ* to show its effect on the coherent dynamics of our system within shorter time scales. The realistic values are indeed much smaller (*γ* ≈ 10^−3^*J* as discussed in the next section) and allows for very high quality transfer even in long chains. Due to the damping dynamics shown in the plot it is wise to only concentrate on the first peak of the fidelity *F*_max_. The latter quantity is displayed in Fig. 6(b) as a function of the dephasing rate *γ* and for different values of *E*. As it is expected *F*_max_ exponentially decays with the increase of *γ*. The decay rate only weakly depends on *E* and slightly become faster for larger *E*.

**Figure 7:**
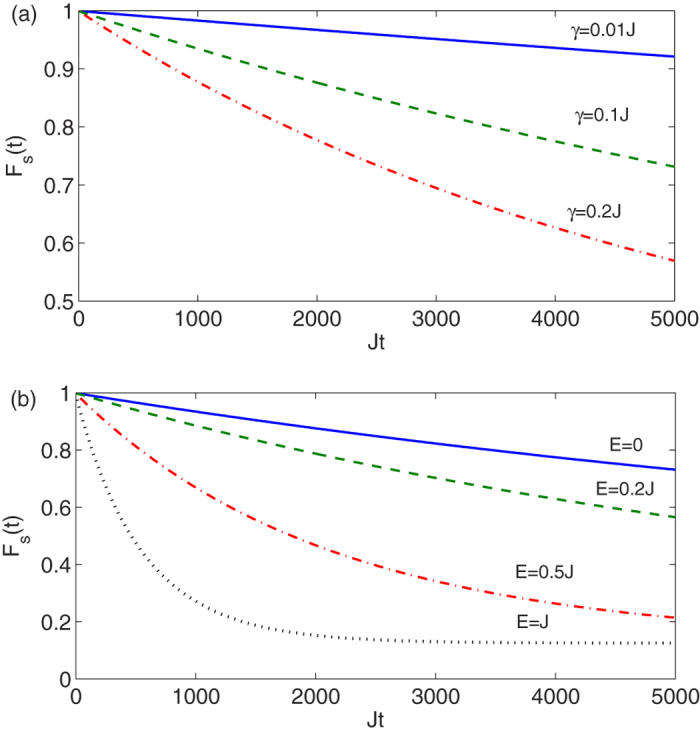
Dynamics in low energy subspace. Storage fidelity *F*_*s*_(*t*) in the memory subspace as a function of time for:(a) Different dephasing rates *γ* and; (b) Different in-plane anisotropy *E*. In both figures *N* = 2 and *D* = −20*J*.

We now study the effect of dephasing on information storage, namely when the system is prepared in the low-energy subspace. To investigate the quality of the storage we define a new fidelity which measures the deviation from the initial state at any time *t*. For example, we consider the initial pure state 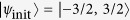 and we define the storage fidelity as 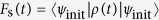 where *ρ*(*t*) is calculated from the master equation (12). In Fig. 7(a) we study the time evolution of the *F*_*s*_(*t*) for different values of *γ*, when *E* = 0. As expected the storage fidelity decays in time with a rate which increases for increasing *γ*. However, within the timescale of tens of operations in the computational subspace (say *Jt* ≈ 100) the quality of the storage is only weakly affected by dephasing, as *F*_s_ remains above 0.95 even for a strong dephasing of *γ* = 0.2*J*.

Unlike the high-energy subspace in which the transmission was hardly affected by the in-plane anisotropy *E*, in the storage subspace the effect is no longer negligible after long times. To show this effect we plot in Fig. 7(b) the storage fidelity as a function of time for different values of *E* when *γ* = 0.5*J*. It turns out that the storage fidelity decays faster for larger values of *E*, due to enhanced coupling of the low and high energy subspaces by the in-plane anisotropy. Since *E* depends on the geometric property of the material, it is preferable to use systems with vanishing *E* to guarantee longer storage times.

It is worth emphasizing that the stability of the low energy subspace with respect to dephasing is significantly enhanced by using larger spins because the effective coupling between neighboring sites exponentially goes down by increasing the spin *S*.

### Long-range interactions

The dipolar or RKKY coupling between nanomagnets may induce interactions beyond the nearest neighbours. To study this effect we modify our Hamiltonian *H*_tot_ in (1) such that two nano-magnets at sites *i* and *j* interact as 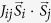, where *J*_*ij*_ = *J*/|*i* − *j*|^3^.

In Table 1 we show the maximum transfer fidelity *F*^max^, and its corresponding transfer time t^*^, in terms of length *N* for both nearest neighbour and long-range interactions. As it is clear from Table 1 the long-range interaction has little effect on the information transfer along the spin chain.

## Implementation

### Molecular magnets

We propose an array of molecular magnets for realizing our theoretical proposal. Indeed, single-molecule magnets are very attractive because of many reasons: (i) they can be prepared by chemical synthesis in a huge range of configurations providing scalability for quantum technology^37^; (ii) they are composed of spin clusters and can be individually addressed because of their large size^38^; (iii) very small ratios of *J*/*D* (≈10^−2^)^27^ have already been realized; (iv) the dissipation time *T*_1_ is extremely large (≈4 ms)^39^, and the decoherence time *T*_2_ exceeds few *μ*s^29^,^30^; (v) the in-plane anisotropy *E* is negligibly small^27^. Molecular rings, such as heterometallic wheels Cr_7_M^29^ (M is a metal center), are promising candidates for realizing our proposed mechanism. By using different metal centers M, one can change the spin sector of the ground state: for instance, *S* = 3/2 is achieved with M = Ru^2+^Ru^3+^ 27. The entanglement properties^40^,^41^ of such rings have been studied and there are proposals to split them into open chains^42^.

Local addressability can be achieved by engineering the *g*-factor in different sites through chemical synthesis^26^,^28^,^43^. This indeed creates site dependent Zeeman energy splitting in the presence of a uniform magnetic field, even without the complexity of a spatially modulated field. Such engineered *g*-factors allows initialization and readout of specific sites using selective microwave pulses which are in resonance only with the target site and practically have no effect on the rest. While the magnetic field is needed for local addressability, as a part of initialization and readout, it should be switched off otherwise.

Initially the system can be prepared in the ferromagnetic state where all the magnets are aligned in the same low-energy quantum state 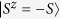 by applying strong magnetic fields^38^,^44^. To write the information in the memory subspace the magnetization of each site can be selectively reversed 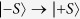 by applying a fast sequence of electromagnetic pulses^45^ or a suitably modulated multi-frequency pulse^46^.

For most of the time, the molecules stay in the low energy subspace, which effectively do not evolve. To transfer information between distant sites one needs to bring all the molecules into the high-energy subspace 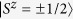 in which the interaction between neighboring sites is strong. Such transitions can be implemented via global electromagnetic pulses which act collectively on all magnets simultaneously. Each pulse makes a magnetic transition with Δ*S*^*z*^ = ±1, till the state is transfered to the high-energy subspace *S*^*z*^ = ±1/2. For instance, for *S* = 3/2 such transition is achieved in a single step. An alternative approach is via using a properly modulated multi-frequency pulse which makes these transitions in a single step^46^. After finishing the transfer, the same set of pulses can be used for bringing the states back to the memory subspace. Since the *g*-factor is site-dependent, as required for local addressability, the transition time for each site will be different. In order to achieve the transition from memory to data-bus subspace (and *vice versa*) with a single global operation, one may use an adiabatic inversion pulse^47^ which is intense and operates within a short period of time. Consequently, this pulse has a wider frequency spectrum capable of exciting all sites in spite of the different resonance energies. There are various ways of implementing such pulses^48^, each one with its specific duration and intensity. For instance, using a linear frequency sweep with range Δ*f*, one requires a pulse duration 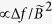 48.

Finally, when the system is back into the memory subspace, thanks to the slow dynamics of the low-energy subspace, there is enough time for readout. Magnetic readout has been experimentally realized with different ways, either with a scanning tunneling microscope (STM)^37^ or with electronic paramagnetic resonance (EPR)^27^,^39^,^45^.

Molecular magnets represent a flexible setup as their magnetic properties can be engineered in a wide range via chemical synthesis. Promising molecules for quantum information applications^27^,^39^ display a small value of *J*/*D* and a large decoherence time. For instance, using heterometallic wheels Cr_7_M^27^, the values *J* ≈ 100 GHz and *D* ≈ 88*J* have been measured.

A typical^39^ *J* = 2 GHz and *D* = −20*J* implies a transmission time of ≈1 ns. Due to the very large *T*_1_ (e.g. ≈1 ms in^39^) the limiting time scale is given by *T*_2_ which exceeds 1*μ*s^27^,^29^,^30^. In our formalism this corresponds to *γ* ≈ 1 M*Hz* and therefore *γ*/*J* ≈ 10^−3^. This allows for ≈10^3^ operations before equilibration. The technology allowing very fast pulse sequences has already been developed^45^ paving the way for controlling the dynamics in the sub-nanosecond regime. This opens the possibility of using molecular clusters with larger exchange interaction *J* allowing even more operations within the coherence time.

### Adatoms on surfaces

Another exciting possibility are structures made from magnetic adatoms (e.g., Co, Mn etc.) created and probed on surfaces using STM^49^. Recently adatomic clusters^7^, and adatoms themselves^8^ on surfaces have indeed been proposed as a quantum storage of a classical bit. Their dephasing has been studied using the same type of weak coupling Lindbladian master equations as considered by us here^8^. This approach has become even more accurate very recently with the advent of superconducting layers replacing the usual two dimensional electron gas in STM so as to greatly increase electron relaxation times for the adatoms^50^. As electron spin scattering is suppressed because of the energy gap of the superconductor, we also naturally expect the dephasing time to be enhanced in addition to the relaxation time. Though the dephasing time is yet to be measured, this kind of work is ultimately aimed towards taking adatoms towards the regime of coherently operating devices. Another technique by which the effective isolation of adatoms has been greatly enhanced is by using symmetry protected systems^10^,^51^. These give the hope that adatoms will eventually approach the coherent regime^51^ so that coherent non-equilibrium dynamics, as used in our paper, will become accessible. Very large anisotropies *D* have also been recently achieved^25^ for Co atoms, for example. Microwaves could still be used for changing between the memory and data-bus modes of the chain, but measurements can be done locally at leisure using spin polarized STMs^9^ after setting the device to memory mode. Moreover, we can bring a magnetic tip close to the adatom (as in the newly devised magnetic exchange force microscopy, which is compatible with STM setup) to apply a local field to it^52^. This local field, if in a pertinent direction, can directly precess the adatom’s spins. Alternatively, it can locally Zeeman split the energy levels so that a microwave can locally flip it. In particular, a more macroscopic nanomagnetic bit attached to the STM tip could be made to talk to the adatom bit by bringing the tip in proximity to it^53^. This may offer a route to interface the system we discussed here with more conventional magnetic memory with larger magnetic bits.

## Conclusions

A general problem in any information processing architecture is that memory cells are supposed to be well decoupled from each other to act as a good information storage, while the data-bus cells should have strong interactions to implement fast quantum gates and information transfer. The different interactions required in the memory and data-bus units make it very challenging to spot a physical system suitable for both, so one way to face this problem is to use hybrid structures^17^,^18^,^19^ which demands very sophisticated control of the system. To avoid such complexities, in this paper we have proposed a mechanism to implement memory and data-bus units, two key requirements for any processor, with the same physical setup, namely arrays of large-spin magnets. The data-bus and memory subspaces are encoded into different spin levels of the same magnet. The selection rules imposed by the exchange coupling together with a large zero field energy splitting result in different effective coupling between spin levels of the two neighboring magnets. While high-energy spin levels are directly coupled by the exchange interaction, lower energy subspaces are coupled only via higher-order processes, which become ineffective in the time scales of operations in the high-energy subspace. Hence, the high-energy subspace is suitable for transmission tasks, while the low-energy subspace can act as a robust memory. Transitions between the two subspaces can be done at will by applying resonant external pulses.

Despite having not gone into the details of how one would engineer gates between the magnetic bits, we would like to point out that bit movement is already a significant step towards it. For example, for other (incoherent) mechanisms of bit movement, gates were immediately accomplished by bringing two bits in close proximity^9^. Of course, in continuation with our coherent bit movement protocol, we would expect that a XOR gate should be implementable for bits brought next to each other (by data-buses) and in memory subspace through their dominantly Ising interaction of Eq. (4)^54^.

The theory that has been developed does not depend on a particular physical realization and can be applied to many systems, such as magnetic adatoms on surfaces^25^, donors on silicon^15^,^16^ and molecular magnets^26^,^27^,^28^,^29^,^30^. The desirable requirements for our scheme are: (i) addressability of some individual magnets to accomplish read/write operations; (ii) long coherence times for transfer over tens of magnetic cells, but not as demanding as for quantum computation; (iii) flexibility in engineering the couplings; (iv) large zero-field energy splitting; and (v) intrinsically vanishing in-plain anisotropy. Our proposed mechanism can be realized in molecular magnets with current technology, and we showed that, using parameters taken from recent experiments, it allows for hundreds of operations within the coherence time of the system.

## Additional Information

**How to cite this article**: Jia, N. *et al.* Integrated information storage and transfer with a coherent magnetic device. *Sci. Rep.* **5**, 13665; doi: 10.1038/srep13665 (2015).

## Supplementary Material

Supplementary Information

## Figures and Tables

**Table 1:** Comparing short-range and long-range interactions.

N	2	3	4	5	6	7	8	9	10
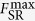	0.9993	0.9829	0.9464	0.9035	0.8764	0.8409	0.8005	0.7776	0.7535
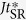	0.79	1.12	1.38	1.73	1.98	2.23	2.47	2.83	3.07
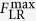	0.9993	0.9805	0.9512	0.9105	0.8653	0.8339	0.8058	0.7753	0.7438
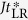	0.79	1.10	1.37	1.62	1.86	2.20	2.43	2.66	2.90

Maximum bit transfer fidelity *F*^max^ and its corresponding transfer time t^*^ as a function of *N* for nearest neighbour, namely short-range (SR), interactions (1) and long-range (LR) interactions.
