# Supplementary material for: Integrated information storage and transfer with a coherent magnetic device
Source: Sci Rep. 2015 Sep 8;5:13665. doi: 10.1038/srep13665 (PMC4561894; doi:10.1038/srep13665)
Supplement: Supplementary Information [file srep13665-s1.pdf]

Supplementary Information: Integrated information storage and transfer with a coherent magnetic device

Ning Jia,[1] Leonardo Banchi,[2] Abolfazl Bayat,[2] Guangjiong Dong,[1] and Sougato Bose[2]

[1]State key laboratory of precision spectroscopy, Department of Physics, East China Normal University, Shanghai 200062, China

[2]Department of Physics and Astronomy, University College London, Gower Street, WC1E 6BT London, United Kingdom

(Dated: February 25, 2015)

Effective Hamiltonian: Partial integration technique

An effective Hamiltonian can be obtained by defining a pair of projection operators, P and Q so that P projects the states into the relevant Hilbert space and $Q = 1 - P$ is a projection to the irrelevant part. Clearly, $P + Q = 1$, $P^2 = P$, $Q^2 = Q$, $PQ = QP = 0$. Inserting some identities $P + Q = 1$ in the Schrödinger equation $i\partial_t |\psi\rangle = H|\psi\rangle$ one obtains

$$i\partial_t P|\psi\rangle = PHP|\psi\rangle + PHQ|\psi\rangle \tag{1}$$

$$i\partial_t Q|\psi\rangle = QHP|\psi\rangle + QHQ|\psi\rangle \tag{2}$$

Then, defining

$$|\psi_P\rangle = P|\psi\rangle \ , \qquad |\psi_Q\rangle = Q|\psi\rangle \ , \tag{3}$$

$$H_P = PHP \ , \quad H_Q = QHQ \ , \quad V = PHQ \ , \tag{4}$$

one obtains

$$i\partial_t |\psi_P\rangle = H_P|\psi_P\rangle + V|\psi_Q\rangle \ , \tag{5}$$

$$i\partial_t |\psi_Q\rangle = V^\dagger|\psi_P\rangle + H_Q|\psi_Q\rangle \ . \tag{6}$$

In a many-spin systems an effective Hamiltonian can be obtained for the memory (low-energy) and data-bus (high-energy) space by defining the projection operators

$$P_{\rm mem} = \sum_{m_i=\pm 3/2} |m_1\rangle\langle m_1| \otimes \cdots \otimes |m_N\rangle\langle m_N| \ , \tag{7}$$

$$P_{\rm bus} = \sum_{m_i=\pm 1/2} |m_1\rangle\langle m_1| \otimes \cdots \otimes |m_N\rangle\langle m_N| \ . \tag{8}$$

Partial integration works when initially $|\psi_Q\rangle$ has no population, and when the relevant and irrelevant subspaces evolve with different characteristic times, i.e. when there is a large energy separation between the eigenvalues of H_Q and those of H_P. In this situation, we use adiabatic partial integration technique to get an approximated formula for $|\psi_Q\rangle$.

We now take transformation, $|\psi_P\rangle = \exp(-iH_P t)|\hat\phi_P\rangle$, and $|\psi_Q\rangle = \exp(-iH_Q t)|\hat\phi_Q\rangle$. From Eqs.(5) and (6) we have

$$i\frac{\partial|\hat\phi_P\rangle}{\partial t} = \hat V(t)|\hat\phi_Q\rangle, \tag{9}$$

$$i\frac{\partial|\hat\phi_Q\rangle}{\partial t} = \hat V(t)^\dagger|\hat\phi_P\rangle. \tag{10}$$

where $\hat V(t) = \exp(iH_P t)V\exp(-iH_Q t)$. Now we diagonalize H_P and H_Q, i.e., $H_P = U_P\lambda_P U_P^\dagger$, $H_Q = U_Q\lambda_Q U_Q^\dagger$, where U_P and U_Q are unitary, λ_P and λ_Q are diagonal with diagonal elements λ_P^i and λ_Q^i. For convenience, we set $|\tilde\phi_Q(t)\rangle = U_Q^\dagger|\hat\phi_Q(t)\rangle$, $|\tilde\phi_P(t)\rangle = U_P^\dagger|\hat\phi_P(t)\rangle$, and $\tilde V(t) = U_P^\dagger\hat V(t)U_Q = e^{i\lambda_P}\tilde V e^{-i\lambda_Q t}$. From Eq. (10), assuming the initial condition $|\hat\phi_Q(0)\rangle = 0$ we have

$$|\tilde\phi_Q(t)\rangle = -i\int_0^t \exp(i\lambda_Q t')\tilde V^\dagger\exp(-i\lambda_P t')|\tilde\phi_P(t)\rangle dt', \tag{11}$$

$$\tilde\phi_{Q,k}(t) = -i\sum_j\int_0^t \tilde V_{jk}^*\exp[i(\lambda_Q^k - \lambda_P^j)t']\tilde\phi_{P,j}(t')dt' \ . \tag{12}$$

where in the second line we used an element wise definition. After a partial integration we have

$$\tilde{\phi}_{Q,k}(t) = -\sum_j \Big(\tilde{W}_{kj}(t)\tilde{\phi}_{P,j}(t) - \tilde{W}_{kj}(0)\tilde{\phi}_{P,j}(0)\Big) + \sum_j \int_0^t \tilde{W}_{kj}(t')\frac{d\tilde{\phi}_{P,j}(t')}{dt'}dt' \tag{13}$$

where $\tilde{W}_{kj}(t) = \tilde{V}^*_{jk}\frac{\exp[i(\lambda^k_Q-\lambda^j_P)t]}{\lambda^k_Q-\lambda^j_P}$. In Eq. (13), the term in the second line is of the order $(\lambda^k_Q - \lambda^j_P)^{-2}$, as it is clear if one continues with partial integrations. When there is a large energy separation, i.e. $|\lambda^k_Q - \lambda^j_P| \gg 1$ and $\tilde{\phi}_{P,j}(t')$ slowly changes with time, one can neglect the second line in (13). Moreover, when also one has $\tilde{W}_{kj}(0)\tilde{\phi}_{P,j}(0) \simeq 0$ then $\tilde{\phi}_{Q,k}(t) \approx -\sum_j \tilde{W}_{kj}(t)\tilde{\phi}_{P,j}(t)$. Therefore

$$|\psi_Q\rangle = -\exp(-iH_Q t)U_Q\tilde{W}(t)U_P^\dagger \exp(iH_P t)\,|\psi_P\rangle \tag{14}$$

$$= -U_Q\tilde{W}U_P^\dagger\,|\psi_P\rangle \equiv -W\,|\psi_P\rangle \tag{15}$$

where $\tilde{W}_{kj} = \frac{\tilde{V}^*_{jk}}{\lambda^k_Q-\lambda^j_P}$ and $W = U_Q\tilde{W}U_P^\dagger$. Therefore one finds

$$i\frac{\partial_t\,|\psi_P\rangle}{\partial t} \approx H_{\text{eff}}\,|\psi_P\rangle\ , \tag{16}$$

where

$$H_{\text{eff}} = H_P - VW\ , \tag{17}$$

and W satisfies

$$H_Q\,W - W\,H_P = V^\dagger\ . \tag{18}$$

In the effective Hamiltonian description, the effects of interactions mediated by virtual populations of the irrelevant energy levels are described by extra effective coupling terms acting on the relevant subspace.

Explicit evaluation of the effective Hamiltonian

Equation (18) can be mapped into a linear equation in an extended Hilbert space

$$R\,\text{vec}(W) = \text{vec}(V^\dagger), \tag{19}$$

$$R = I_P \otimes H_Q - H_P^T \otimes I_Q \tag{20}$$

where I_j is an identity matrix which has the same dimensionality of matrix H_j, and $\text{vec}X$ is the column vector obtained by stacking the columns of the matrix X on top of one another. Let us suppose that R can be decomposed into a large and a small part, i.e. $R = R_0 + R'$ where the eigenvalues of R_0 are much larger than those of R'. In this paper we concentrate on the Hamiltonian (**??**) when $D \gg E, J$, so the large part R_0 is proportional to D, $R_0 = DM$, while M and R' are independent on D.

The operator W can be evaluated perturbatively when R is non-singular

$$\text{vec}(W) = R^{-1}\,\text{vec}(V^\dagger), \tag{21}$$

where

$$R^{-1} = \tfrac{1}{DM+R'} = \tfrac{1}{1+(DM)^{-1}R'}\tfrac{1}{DM} = \tfrac{1}{DM} - \tfrac{1}{DM}R'\tfrac{1}{DM} + \tfrac{1}{DM}R'\tfrac{1}{DM}R'\tfrac{1}{DM} + \dots \tag{22}$$

An effective Hamiltonian valid to a particular order n in $\frac{1}{D}$ is obtained by truncating the above infinite series to the nth order.
